# A rapid and noninvasive method to detect dried saliva stains from human skin using fluorescent spectroscopy

**DOI:** 10.4103/0973-029X.80033

**Published:** 2011

**Authors:** Kanwar Deep Singh Nanda, K Ranganathan, KM Umadevi, Elizabeth Joshua

**Affiliations:** *Department of Oral pathology and Microbiology, Ragas Dental College, Chennai, Tamil Nadu, India*

**Keywords:** Fluorescence, forensic dentistry, saliva, spectroscopy, tryptophan

## Abstract

**Objective:**

Saliva is one of the vital fluids secreted in human beings. Significant amount of saliva is deposited on the skin during biting, sucking or licking, and can act as an important source in forensic evidence. An enzyme, α amylase, gives a characteristic emission spectrum at 345–355 nm when excited at 282 nm and this can be identified by using fluorescent spectroscopy and can help in forensic identification. This study describes a rapid method to detect dried saliva on the human skin by fluorescent spectroscopy.

**Materials and Methods:**

This study included 10 volunteers, who deposited their own saliva on skin of their ventral forearm by licking and water on the contralateral arm as control. This study was carried out at Central Leather Research Institute, Chennai.

**Study design:**

Ten volunteers deposited their own saliva on skin of their ventral forearm by licking. A control sample of water was deposited at the contralateral arm. Each sample was excited at 282 nm and emission spectrum was recorded.

**Results:**

The emission spectra of 10 swab samples taken from dried saliva were characterized at the primary peak of 345 to 355 nm whereas the emission spectrum of water as a control was recorded at 362 nm.

**Conclusion:**

The presence of emission spectrum at 345–355 nm with excitation at 282 nm proves to be a strong indicator of saliva deposited on human skin.

## INTRODUCTION

In forensic cases of sexual assault and child abuse, bite marks analysis is very difficult because human dentition does not always leave identifying features imprinted on the skin surface.[1] Saliva is one of the vital fluids secreted in human beings, which is deposited on the human skin through biting, sucking, licking, kissing, and possibly through other behaviors.[2,3] Detection of saliva stains encountered in forensic science casework is one of the primary objectives for forensic serologist as saliva is an important source of DNA.[4] Detection of saliva from human skin can be an important source for identifying an individual. Unfortunately, dried saliva stains are invisible to the human eye, which adds to difficulty of recognizing and collecting. The DNA present in saliva on skin is difficult to collect and extract than similar stains on clothing, paper or other inanimate objects since substrate on which saliva is deposited (skin) cannot be submitted directly to extraction procedures. Therefore, an improved collection method is required first to identify the invisible saliva stains on human skin and then proceed with other methods of extracting DNA to identify the suspect and exclude the innocent.[5] Various methods for detecting dried saliva stains have been tried out like use of chemicals, lasers and fluorescence, but each test has its own limitations.[6]

Fluorescence spectroscopy is noninvasive, having a high sensitivity and selectivity which allows measurement under physiological conditions and is cost effective (approximately 1400 per sample). This makes fluorescent spectroscopy a real-time diagnostic technique in the field of forensic science.[7]

## MATERIALS AND METHODS

This study was divided into three phases.

- Determination of optimum excitation wavelength of undiluted saliva
  Undiluted saliva from two volunteers was excited with a wavelength between 200 and 320 nm. The peak excitation wavelength was used to obtain the emission spectrum for the dried saliva sample collected.
- Fluorescence spectroscopy of saliva and control samples from skin
  Ten volunteers deposited their own saliva on the marked area of their forearm by licking at normal room temperature in the morning. Before depositing saliva, the forearm was cleaned with soap and dried to prevent any source of contamination. As a control, water was deposited on the forearm of the opposite side. Both saliva and water were allowed to air dry for 30-45 minutes. A fiber-free cotton dipped in pH 7.4 phosphate buffer 0.1 M KCl with an excess solution removed was rubbed over the marked area. Second swab was taken from the control site of the opposite arm. Each swab was mixed in a separate cuvette containing 2 ml KCl solution for 10 seconds. Finally, the contents of each cuvette were then transferred to a quartz cuvette and the fluorescence emission spectrum was recorded from 300 to 540 nm using a spectrofluorimeter.
- Fluorescence spectroscopy of tryptophan
  The emission spectrum of tryptophan was recorded by dissolving 0.5 mg/ml of tryptophan in 5 mM KCl. This solution was excited at a wavelength of 282 nm. The emission spectrum obtained was compared with the emission spectrum obtained from 10 volunteers’ saliva samples.

## RESULTS

**Figure 1:**
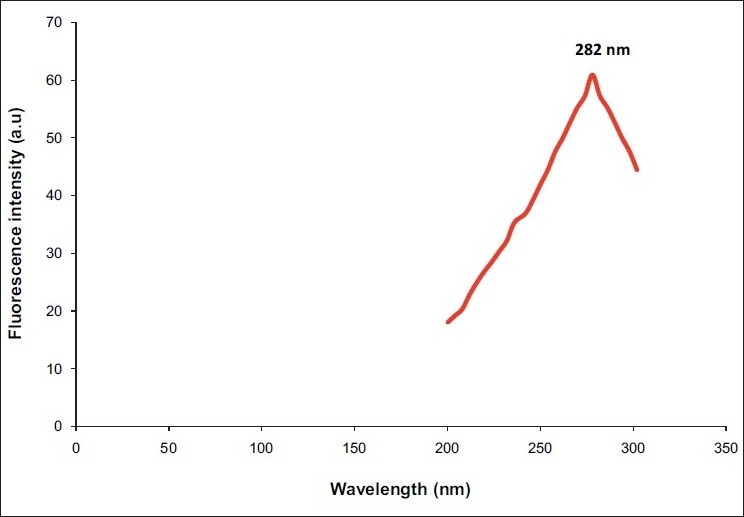
Absorption spectra of undiluted saliva sample

**Figure 2:**
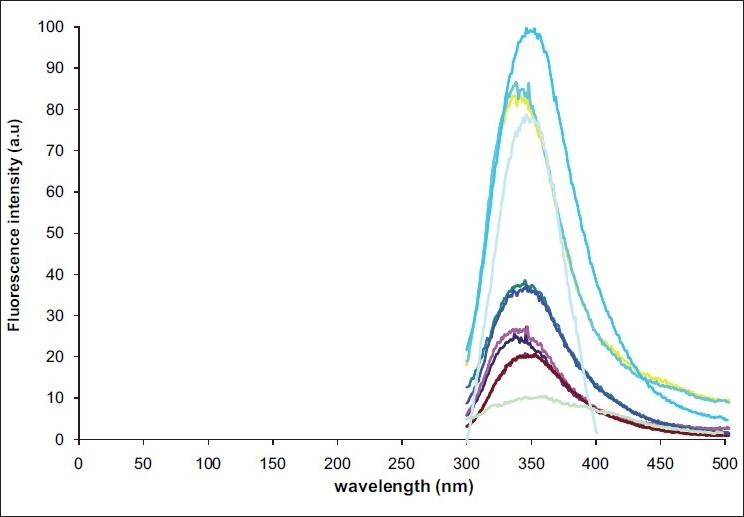
Emission spectra and fluorescence intensity of saliva and control samples

**Figure 3:**
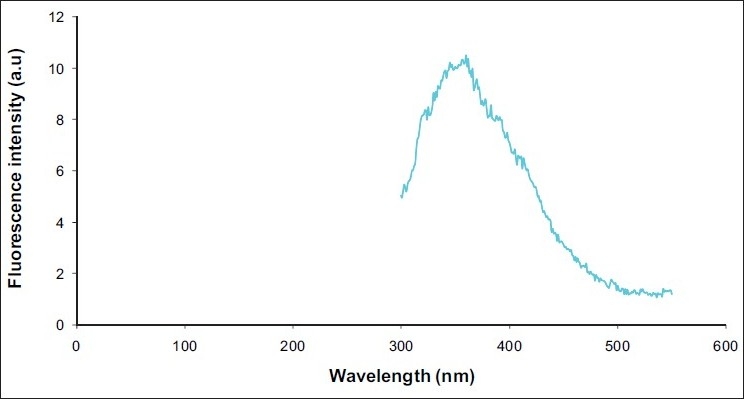
Emission spectrum of water as a control

**Figure 4:**
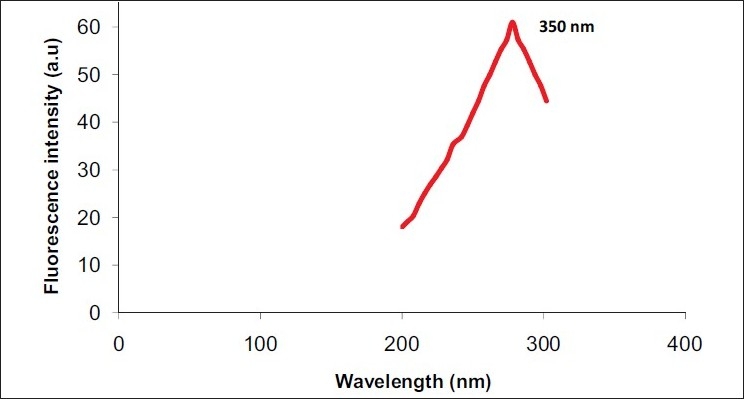
Emission spectra of tryptophan

- Absorption spectra of undiluted saliva samples
  The maximum absorption spectra of undiluted liquid saliva samples were characterized by an excitation peak at 282 nm [Figure 1] which was considered as the maximum excitation wavelength for obtaining emission scan of swab contents.
- Emission spectra and fluorescence intensity of saliva and control samples
  The emission spectra of 10 swab samples taken from dried saliva were characterized at the primary peak of 345 to 355 nm [Figure 2], whereas the emission spectrum of water as a control was recorded at 362 nm [Figure 3].
- Emission spectra of tryptophan
  The peak emission spectrum of tryptophan was recorded at 350 nm [Figure 4] which matched well with the emission spectrum obtained from 10 saliva swab contents from human skin.

## DISCUSSION

There are many procedures applied in detecting dried saliva, such as use of various lights and chemicals; but due to the limitations of each test, they are not able to match the efficiency and rapid nature of fluorescent spectroscopy.[8] Researches in the field of biophysics after fluorescent spectroscopy have brought revolution in the field of forensic science. Fluorescence spectroscopy is widely used to analyze structure, dynamics and functional interactions of proteins.[7] It is based on the principle that when a fluorescent material is excited at a particular wavelength, it emits radiation of longer wavelength which can be recorded.[9] The aromatic amino acid, tryptophan, which is one of the important amino acids in α salivary amylase, an enzyme present in saliva, gives a characteristic emission spectrum at 345–355 nm when excited at a particular wavelength of 282 nm.[10]

The bands obtained from samples of dried saliva when analyzed with fluorescence spectroscopy conformed well to those obtained from pure tryptophan. This proved well that the swab samples collected were of saliva. The peak of fluorescence intensity in saliva was found to vary among the 10 volunteers, which may be due to different protein content of saliva from each individual [Figure 2].

Use of various light sources like UV light and laser has been suggested as a simple screening technique in identifying stains of body fluids like dried saliva, but they were detected in only 13 and 21% of cases, respectively.[6] Similarly, use of quartz arch tube and argon ion laser has been tried in detecting dried saliva stains and proved to be useful in only 48 and 30% of cases, respectively,[11] as compared to 100% in the present study. Short UV luminescence using Nd:YAG laser emitting at 266 nm has also been tried in detecting saliva stains invisible to the naked eye in a preliminary study, which has certain disadvantages like risk of burning one’s hand, conjunctivitis and lack of portability.[8] Recent studies done by the same research group concerning the use of mercury xenon lamp and CCD camera for detection of the fluorescence of different body fluids including saliva did not show any clear data.[12]

Various chemicals like enzymes and salts have also been tried out to detect dried saliva stains. Most commonly used enzymes are alkaline phosphatase, starch and amylase.[13–15] Unfortunately, there are limitations of each test; alkaline phosphatase is not very specific as it gives a false-positive result.[13] Starch or iodine test for amylase has been used for many years, but the major limitation is that excess of starch gives a negative reaction which leads to false-positive result.[14] The Phadebas amylase[15] test has a main disadvantage that amylase only above a certain limit of 0.02 units can be regarded as a strong indicator of the presence of saliva and no clear threshold has been defined for detecting amylase.[16,17]

Using salts like nitrate and thiocyanate has been tried, but the limitation with nitrate is that this test is applicable to recent samples of 2 days only, whereas thiocyanate test is not always present in saliva.[13]

Fluorescent spectroscopy has a good sensitivity in detecting dried saliva stains on skin.[3] It can be a useful tool for forensic examiners who face problem in cases of bite marks’ analysis because human dentition does not leave identifying features imprinted on the skin surface.[1] Other advantages are that same sample can be used for DNA analysis after fluorescence measurement[10] and the time required for the whole procedure is less than 10 minutes. From a practical point of view, this technique could detect saliva in samples obtained from skin area of suspicion, but if we do not know the exact site of dried saliva deposition, then to scan quickly a large area of body, laser and fiber-based instruments can be used as an adjunct to fluorescence spectroscopy.[7,8,12]

The results of our study suggest that tryptophan can act as one of the prevalent probes in dried saliva stains on human skin for fluorescence analysis and can be used for the detection of saliva in forensic cases. Larger sample size will help us to better define the usefulness of fluorescence spectroscopy as a diagnostic tool.

## CONCLUSION

Fluorescence spectroscopy is a rapid, sensitive and noninvasive technique for the detection of dried saliva stains on skin. This method, which has mainly been used for diagnostic application, could significantly contribute to forensic science.
